# The Rapid Progression of Myelopathy Due to Cervical Epidural Fluid Collection From Metastatic Tumor in the Cervical Lamina: A Case Report

**DOI:** 10.7759/cureus.78787

**Published:** 2025-02-09

**Authors:** Eitaro Okumura, Kotaro Kohara, Maegawa Tatsuya, Ryo Hashimoto, Motoo Kubota

**Affiliations:** 1 Department of Spine Surgery, Kameda Medical Center, Chiba, JPN

**Keywords:** cervical compression myelopathy, cervical epidural fluid collection, lung cancer, metastatic spinal tumor, rapid accumulation

## Abstract

Various conditions can cause myelopathy due to cervical epidural fluid collection, including idiopathic cervical epidural hematoma, traumatic cervical epidural hematoma, infectious myelitis, epidural abscess, spinal cord infarction, post-traumatic cerebrospinal fluid (CSF) leakage, and epidural tumors. While physical compression from hematoma, abscess, or epidural tumors is common, and carcinomatous meningitis can cause CSF flow obstruction and accumulation leading to myelopathy, rapid progression of serous fluid collection causing myelopathy is rare. We report a case of myelopathy caused by rapid accumulation of epidural exudate from a metastatic tumor in the cervical lamina.

A 59-year-old male with a history of lung cancer with metastasis to the C3 lamina, who was previously independent in activities of daily living, presented to the emergency department with progressive quadriparesis and urinary dysfunction after farming work. An acute cervical epidural hematoma was initially diagnosed, and emergency surgery was subsequently performed. Intraoperatively, no clear epidural hematoma was found, but serous, light yellow, clear fluid collection was observed in the epidural space. After drainage and partial C3, C6 laminectomy and complete C4, C5 laminectomy for decompression, neurological symptoms improved significantly. Postoperative spinal myelography showed no evident CSF leakage into the cervical epidural space. However, on postoperative day 20, bilateral lower limb weakness recurred with more fluid accumulation than preoperatively. During reoperation, exudate was observed from the remaining portion of the C3 lamina with known lung cancer metastasis. Believing the spinal cord compression from this fluid collection to be the cause of myelopathy, the metastatic C3 lamina was completely removed to prevent recurrence. No obvious dural fistula was observed. After reoperation, no significant epidural fluid collection causing spinal cord compression was observed, and the patient was discharged home with a modified Rankin scale score of 4.

## Introduction

Cervical epidural fluid collection leading to myelopathy can result from various conditions, including vascular (idiopathic cervical epidural hematoma, traumatic cervical epidural hematoma, spinal arteriovenous malformation, spinal cord infarction), infectious (epidural abscess, infectious spondylitis, infectious discitis), neoplastic (epidural tumors, spinal cord tumors), autoimmune, post-traumatic cerebrospinal fluid (CSF) leakage, and iatrogenic causes [1-4]. In clinical practice, the most common cervical epidural fluid is hematoma. While spinal cord compression can occur from viscous fluids like hematoma or abscess, solid masses like tumors, or CSF accumulation due to carcinomatous meningitis, rapid progression of serous fluid collection causing myelopathy is rare. We report a case of myelopathy caused by rapid accumulation of epidural exudate from a metastatic tumor in the cervical lamina.

## Case presentation

The patient was a 59-year-old male diagnosed with lung adenocarcinoma, staged as cT1bN2M0 Stage IIIA. Chemotherapy had been initiated 10 years ago, and a total of 10 different anticancer drugs had been administered, with the tumor showing cycles of growth and reduction. Complete remission had not been achieved, and cervical spine metastasis had been detected two years ago. Radiation therapy (20Gy/5Fr) had been performed for cervical metastasis.

**Figure 1:**
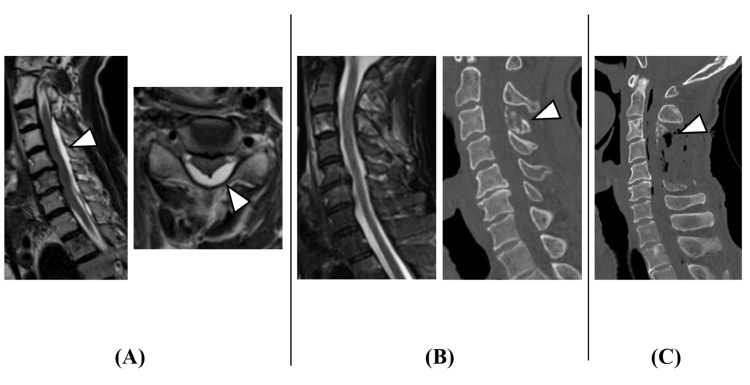
Cervical images (A) Cervical spine MRI at onset (T2-weighted sagittal image and axial image). Due to the acute onset, cervical epidural hematoma was initially suspected (arrowheads). (B) Cervical spine MRI (T2-weighted sagittal image) and CT (sagittal image) obtained two months before symptom onset. There was no obvious epidural effusion. The C3 lamina suggested bone metastasis from lung cancer (arrowhead). (C) Postoperative cervical spine CT (sagittal image). No obvious cervical epidural hematoma was observed intraoperatively. However, serous light yellow effusion was noted. The C3 lamina, which had bone metastasis from lung cancer, was partially resected (arrowhead) CT: computed tomography; MRI: magnetic resonance imaging

**Figure 2:**
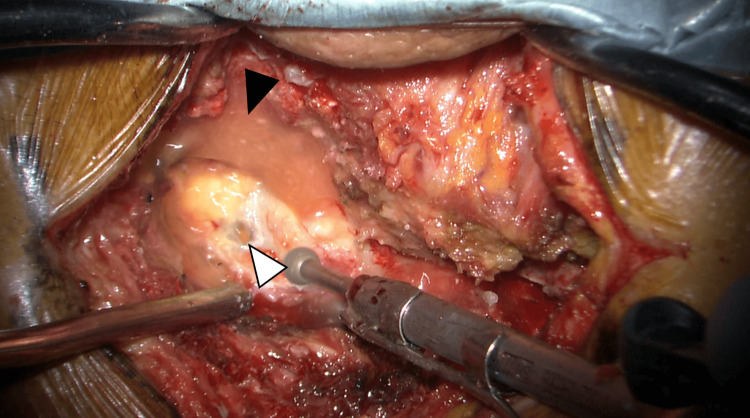
Intraoperative findings The left side of the picture is the cranial side; the right side is the caudal side; the top is the right side; and the bottom is the left side. A midline longitudinal incision was made over the posterior cervical spine. During drilling, when a partial hole was made in the ligamentum flavum, light yellow serous fluid spurted from the C3 epidural space (white arrowhead). The fluid accumulation was also observed (black arrowhead)

The patient had experienced sudden neck pain during farming work, followed by progressive quadriparesis and urinary dysfunction, and he presented to our emergency department three days after the onset of neck pain. Although he could walk with assistance at presentation, his symptoms worsened during the examination, and he developed quadriparesis [manual muscle testing (MMT) grading: 1/5], bilateral hand sensory disturbance, and urinary retention. Emergency MRI revealed cervical epidural fluid collection with spinal cord compression (Figure 1A). Since no evident epidural fluid collection had been seen on cervical MRI taken two months prior (Figure 1B), acute cervical epidural hematoma was suspected, and emergency surgery was performed. However, intraoperative findings showed no clear hematoma but light yellow serous exudate in the epidural space (Figure 2). After drainage and partial C3 laminectomy, complete C4,5 laminectomy, and partial C6 laminectomy for decompression (Figure 1C), the intradural space was examined but appeared normal without hematoma.

**Figure 3:**
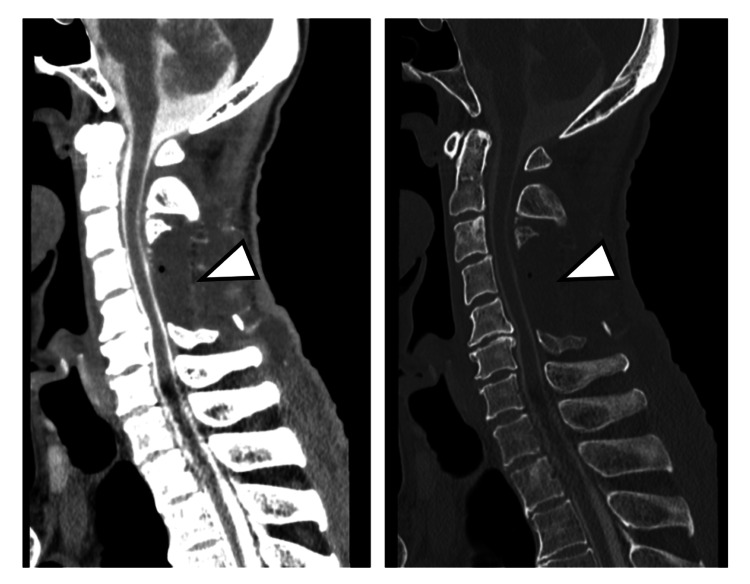
Cervical myelography CT (sagittal image) obtained 10 days after operation No apparent cervical epidural contrast leakage was observed (arrowhead) CT: computed tomography

In the immediate postoperative period, the patient achieved full muscle strength (MMT grading: 5/5) in all extremities, with only mild weakness (MMT grading: 4/5) remaining in the right flexor digitorum profundus and right abductor digiti minimi. Additionally, the patient recovered the ability to walk independently. Spinal myelography on postoperative day 10 showed no evident arachnoid cyst or CSF leakage at the cervical level (Figure 3).

**Figure 4:**
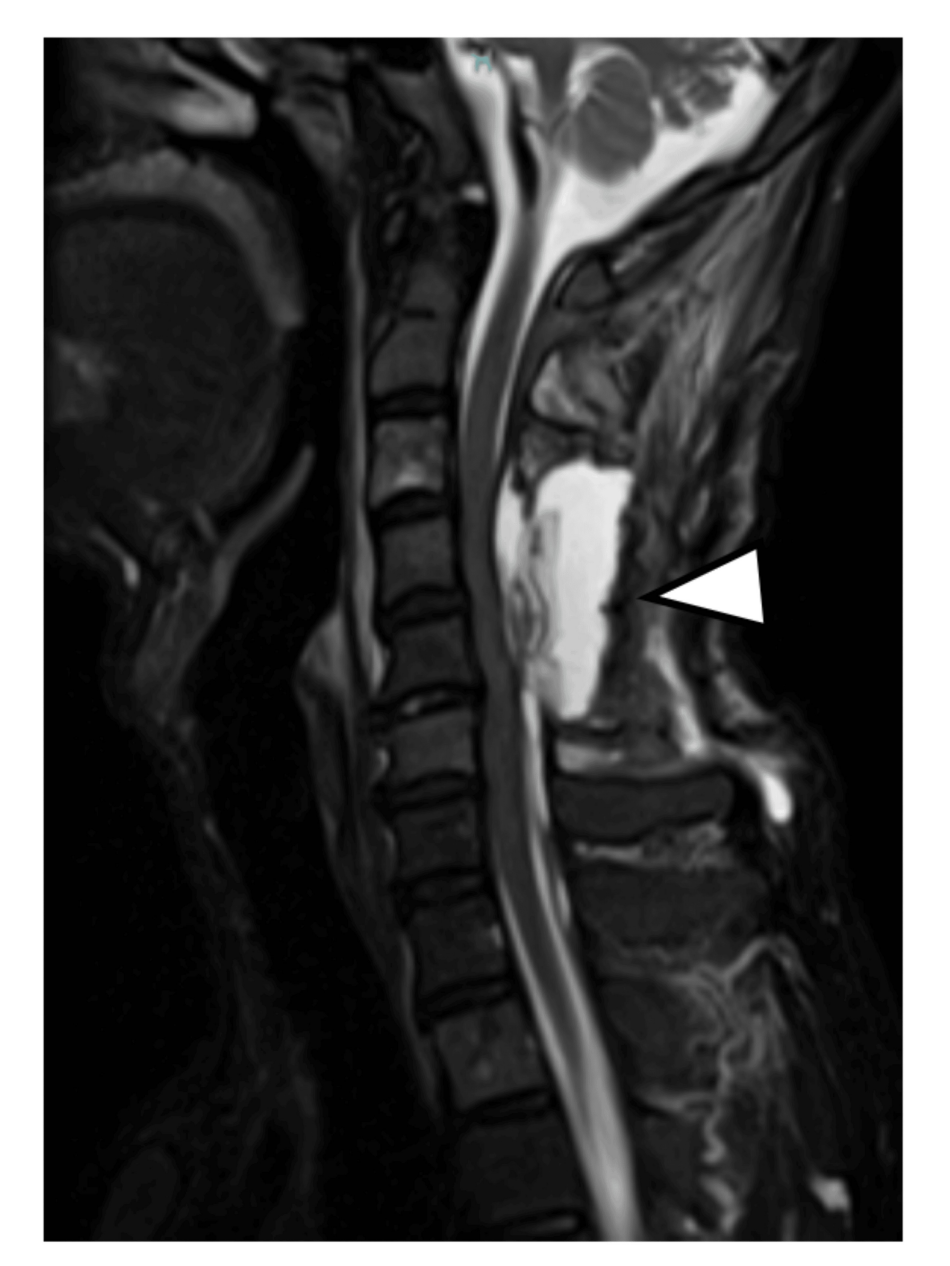
Cervical spine MRI (T2-weighted sagittal image) obtained 20 days after operation Recurrent retained effusion and cervical cord compression were observed (arrowhead) MRI: magnetic resonance imaging

However, on postoperative day 20, the patient developed acute bilateral lower extremity weakness (MMT grading: 4/5), accompanied by weakness in the bilateral distal upper extremities (MMT grading: 3/5). Although no pathological reflexes were observed, the patellar and Achilles tendon reflexes were normal, whereas the biceps, brachioradialis, and triceps reflexes were hyperactive bilaterally. Cervical MRI showed more fluid collection than during the initial surgery (Figure 4).

**Figure 5:**
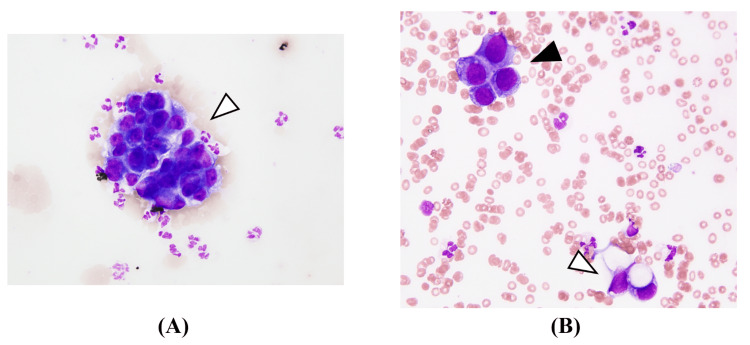
Cytological findings (A) and (B) show cytological findings from the cervical epidural fluid and cerebrospinal fluid, respectively. The epidural specimen (A) demonstrates hypercellular, sanguineous fluid containing spheroid clusters of adenocarcinoma cells, characterized by large nuclei, distinct nucleoli, and cuboid cytoplasm with high N/C ratios (arrowhead in A). The cerebrospinal fluid specimen (B) reveals similar adenocarcinoma cell clusters with hyperchromatic nuclei (black arrowhead in B) and vacuolated cytoplasm, amid numerous neutrophils and histiocytes (white arrowhead in B). Both specimens are consistent with metastatic non-mucinous lung adenocarcinoma

A sample of the fluid accumulated in the cervical epidural space was obtained by aspiration and submitted for pathological examination. Similarly, a CSF sample was also collected and submitted for pathological analysis. These examinations revealed numerous atypical cells possessing large hyperchromatic nuclei and vacuolated cytoplasm in the collected fluid (Figure 5).

**Figure 6:**
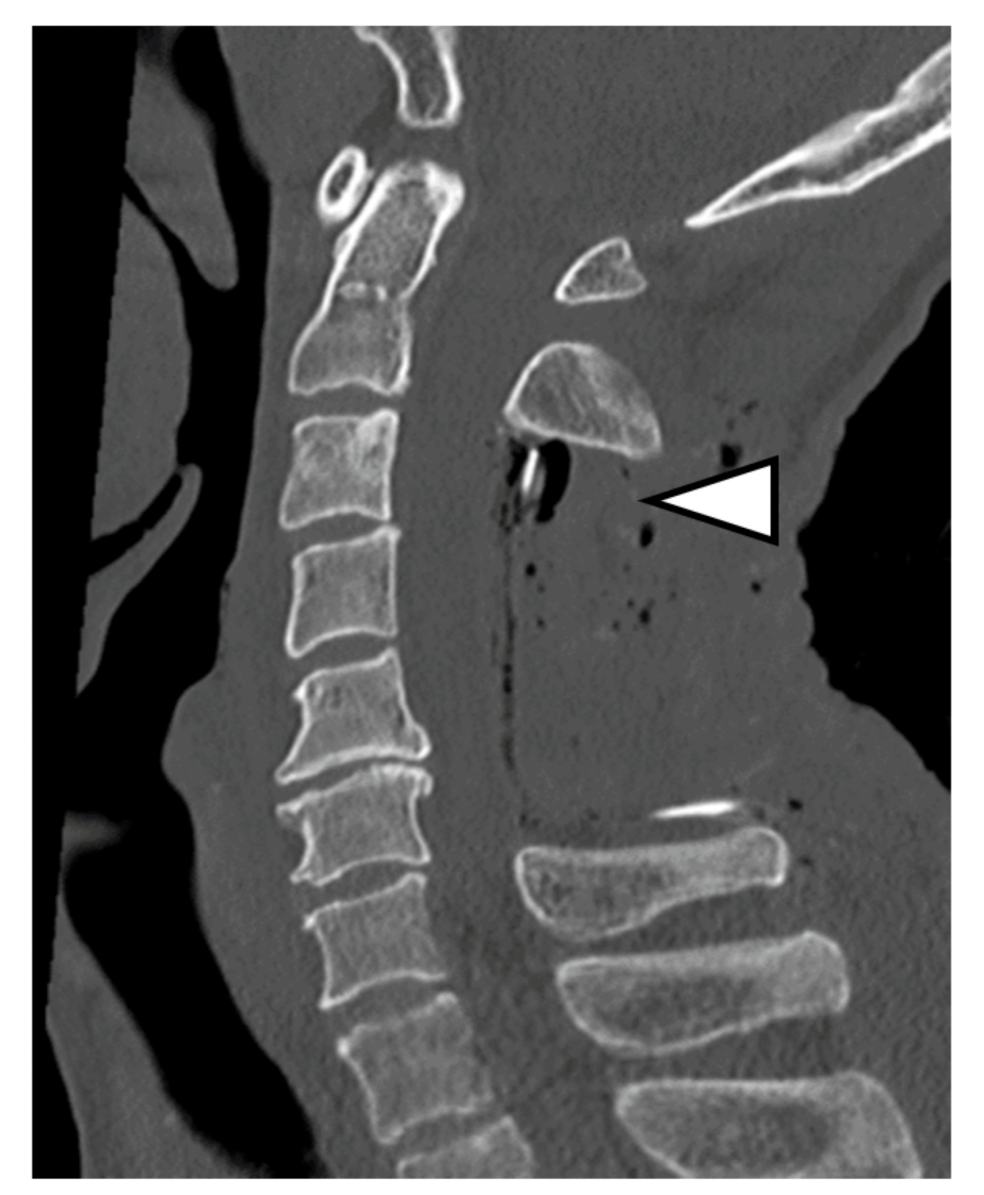
Post-reoperative cervical spine CT (sagittal image) The C3 lamina was completely resected (arrowhead) CT: computed tomography

During reoperation on postoperative day 21, minimal exudate leakage was observed from the C3 lamina with lung cancer metastasis. Believing this accumulation and resulting spinal cord compression to be the cause, the metastatic C3 lamina was completely removed (Figure 6).

No obvious dural fistula was found intraoperatively, and no significant spinal cord compression recurred after reoperation. Muscle strength in both lower limbs improved to MMT grading 4/5; however, the patient was unsteady and could only walk with light assistance. He was discharged home 29 days after reoperation with a modified Rankin scale score of 4.

## Discussion

Spontaneous spinal epidural hematoma is a condition causing radiating pain from the hematoma site followed by motor paralysis and sensory disturbance, with an incidence of 0.1 per 100,000 people annually [5]. While some cases show symptom progression after 48 hours, most progress within 12 hours [6]. In this case, the three-day progression from neck pain to quadriparesis was atypical for spontaneous epidural hematoma. Intraoperative findings showed only light yellow exudate without hematoma, ruling out spontaneous spinal epidural hematoma. While direct spinal cord compression from epidural metastasis or intramedullary metastasis can cause myelopathy [7,8], symptoms typically progress gradually. In this case, no epidural fluid collection had been seen on the cervical MRI two months prior. The rapid progression of symptoms and fluid accumulation after farming work might be attributable to minimal pathological fracture of the metastatic C3 lamina from neck strain while using a grass cutter, promoting exudate accumulation, or tumor progression.

**Figure 7:**
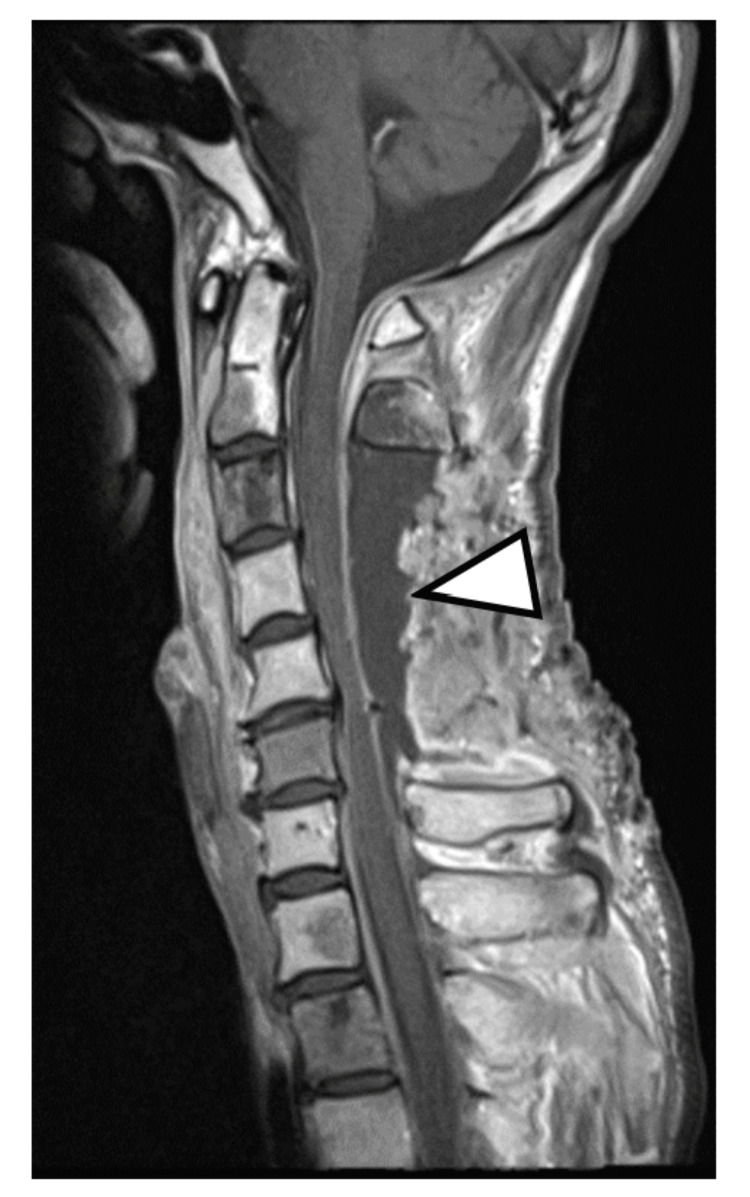
Contrast-enhanced cervical spine MRI (T1-weighted sagittal image) obtained 27 days after reoperation Contrast enhancement of the cervical dura was observed, suggesting the dissemination of cancer cells in the cerebrospinal fluid. No obvious spinal cord compression was noted (arrowhead) MRI: magnetic resonance imaging

After cervical laminectomy, exudate accumulated in the decompressed area, causing recurrent weakness. Reoperation revealed exudate seeping from the C3 laminar metastasis site. Complete removal of the metastatic C3 lamina during initial surgery might have prevented reoperation. Since there was neither an apparent dural fistula nor an arachnoid cyst, considering the results of cytology in the cervical epidural fluid, we determined that the cervical cord compression was caused by exudate accumulation from the metastatic C3 lamina. Post-reoperation contrast-enhanced cervical MRI showed dural thickening and enhancement, suggesting carcinomatous meningitis and terminal cancer status based on the results of cytology in the CSF (Figure 7). While tumor cells are known to show local proliferation as in cortical metastasis or epidural bone metastasis [9], spinal cord compression from fluid component accumulation is extremely rare.

## Conclusions

We presented a rare case of myelopathy caused by rapid accumulation of cervical epidural exudate from a metastatic tumor in the cervical lamina. Despite initial surgical intervention, fluid reaccumulation occurred, necessitating a second surgery involving the complete removal of the metastatic lamina. The patient's neurological symptoms improved following the second procedure, underscoring the complexity of managing such unusual metastatic complications.
